# Impact of bacterial sexually transmitted infections on human papillomavirus and anal dysplasia in people living with HIV

**DOI:** 10.1038/s41598-026-47551-1

**Published:** 2026-04-09

**Authors:** Aroa Villoslada, Adrian Rodriguez, Patricia Sorni, Araceli Serrano, Andrea Salom, Carmen Collado, Mercedes García-Gasalla, Antoni Payeras

**Affiliations:** 1 Internal Medicine (Infectious Diseases), Hospital Universitari Son Llàtzer, 07198 Palma, Spain; 2 Health Research Institute of the Balearic Islands (IdISBa), 07010 Palma, Spain; 3 Faculty of Medicine, University of the Balearic Islands (UIB), Ctra. Valldemossa, km 7.5, 07122 Palma, Spain; 4 Microbiology Service, Hospital Universitari Son Llàtzer, 07198 Palma, Spain; 5 Internal Medicine (Infectious Diseases), Hospital Universitari Son Espases, 07198 Palma, Spain; 6 Centro de Investigación Biomédica en Red, Enfermedades Infecciosas (CIBERINFEC), Av. Monforte de Lemos 3-5, 28029 Madrid, Spain

**Keywords:** HPV, STIs, PLHIV, Anal dysplasia, Screening, Cancer, Diseases, Medical research, Microbiology, Oncology

## Abstract

Anal carcinoma is a relevant malignancy with increased incidence among people living with HIV (PLHIV), particularly in men who have sex with men (MSM) and transgender women (TW). Its main precursor lesion, high-grade anal intraepithelial neoplasia (HGAIN), is primarily associated with persistent infection with human papillomavirus (HPV). However, bacterial sexually transmitted infections (STIs) have been proposed as potential cofactors in anal carcinogenesis. The objectives of this study are to evaluate the relationship between asymptomatic bacterial anal STIs, the presence of HGAIN, and the diversity of oncogenic HPV genotypes in a cohort of MSM and TW living with HIV. A prospective study (June 2017–December 2023) was conducted at Son Llàtzer University Hospital (Palma de Mallorca, Spain), including 377 PLHIV (370 MSM, 7 TW). Anal cytology, detection of 14 oncogenic HPV genotypes, and screening for *Chlamydia trachomatis*,* Neisseria gonorrhoeae*,* and Mycoplasma genitalium* (from 2020 onward) were performed. Participants with abnormal cytology and/or HPV-16/18 underwent high-resolution anoscopy and biopsy. A total of 715 screening procedures were performed. Mean age was 44 years; 89% had an undetectable HIV viral load. The prevalence of bacterial STIs was 5.9% (*N* = 42/715) for *Chlamydia trachomatis*, 2.8% (*N* = 20/715) for *Neisseria gonorrhoeae*, and 10.3% (*N* = 45/438) for *Mycoplasma genitalium*. Oncogenic HPV was detected in 62.6% of samples, with HPV-16 being the most frequent genotype. Participants with bacterial STIs showed greater diversity of oncogenic HPV genotypes, particularly the association of *C. trachomatis* with HPV-16 and HPV-59, and *N. gonorrhoeae* with HPV-18, HPV-33 and HPV-68. Although no statistically significant differences in HGAIN rates were observed, individuals with bacterial STIs and high-risk HPV showed a numerically higher proportion of abnormal cytology. Asymptomatic bacterial anal STIs were common among MSM and TW living with HIV and were associated with greater oncogenic HPV diversity. Although no statistically significant association with HGAIN was demonstrated, the observed pattern may indicate that bacterial STIs influence HPV genotype distribution. Given the established role of specific high-risk genotypes, particularly HPV-16, in anal carcinogenesis, alterations in genotype distribution could theoretically contribute to carcinogenic pathways; however, no causal inference can be made, and longitudinal studies are needed to clarify their potential clinical implications. In this context, integrating bacterial STI screening into anal dysplasia surveillance programs may contribute to a more comprehensive approach to sexual health care in PLHIV.

## Introduction

Anal carcinoma is a relevant malignancy with increased incidence among people living with HIV (PLHIV), with a particularly high impact in men who have sex with men (MSM) and transgender women (TW)^1–5^. High-grade anal intraepithelial neoplasia (HGAIN) represents the most significant precursor lesion of this tumor, and its main etiologic factor is persistent infection by oncogenic genotypes of human papillomavirus (HPV), particularly HPV-16^1–4^.

However, the mere presence of HPV-16 does not fully explain the progression to anal carcinoma, suggesting the participation of additional cofactors in carcinogenesis^5–7^. In the context of cervical cancer, other sexually transmitted infections (STIs), such as *Chlamydia trachomatis*, have been shown to promote a chronic inflammatory state that may enhance HPV-induced lesion progression^8–10^.

Among MSM living with HIV, the prevalence of anal STIs, often presenting as proctitis, is high^11^. Nevertheless, the interaction between these infections and the development of HGAIN remains poorly explored, and the available results are inconsistent. Recent studies have suggested that certain bacterial STIs—particularly *Chlamydia trachomatis* and *Neisseria gonorrhoeae*—may influence the diversity and persistence of HPV genotypes, thereby increasing the risk of high-grade lesions^12,13^.

In 2017, our center implemented a systematic HGAIN screening program including anal cytology and detection of STIs. In this context, the objective of the present study was to evaluate the relationship between asymptomatic bacterial anal STIs, the presence of HGAIN, and the distribution of oncogenic HPV genotypes in a cohort of MSM and TW living with HIV.

## Methods

### Study design and population

This was a prospective study conducted between June 1, 2017, and December 31, 2023, including all PLVIH aged ≥ 18 years—MSM or TW—who attended the Infectious Diseases Unit of Son Llàtzer University Hospital (Palma de Mallorca, Spain) and were undergoing screening for HGAIN. Anal dysplasia screening was systematically offered to all MSM and TW living with HIV who were followed at our unit as part of the institutional clinical protocol. Participation in the screening program was voluntary.

All participants were enrolled in the Balearic eVIHA cohort, a regional clinical platform used in all hospitals of the Balearic Islands for longitudinal follow-up of PLHIV. Written informed consent was obtained from all participants prior to inclusion. The methodological, ethical, and legal aspects of the eVIHA protocol (code IB 3808/18 PI) were approved by the Research Ethics Committee of the Balearic Islands, in accordance with the ethical standards of the institutional and national research committees and with the 1964 Declaration of Helsinki and its subsequent amendments.

Baseline evaluation included anal examination, cytology, and polymerase chain reaction (PCR) testing for 14 oncogenic HPV genotypes. Screening for bacterial STIs—including *Chlamydia trachomatis*, *Neisseria gonorrhoeae*, and *Mycoplasma genitalium*—was performed using anal swabs. Detection of *M. genitalium* was incorporated into the protocol from 2020 onward.

### Sample collection and laboratory procedures

At each baseline and follow-up visit, two anal swabs were collected using Dacron applicators:

1. One specimen was placed in a ThinPrep liquid-based cytology medium and processed by the Pathology Department.
2. The second specimen was sent to the Microbiology Department for detection of high-risk HPV (HR-HPV), *C. trachomatis*, *N. gonorrhoeae*, and *M. genitalium* by PCR.

Cytological samples were evaluated by expert pathologists and classified according to the Bethesda System^14^ as: negative, ASCUS (atypical squamous cells of undetermined significance), LSIL (low-grade squamous intraepithelial lesion), HSIL (high-grade squamous intraepithelial lesion), or anal squamous cell carcinoma (SCC).

Detection of HR-HPV was performed using the Anyplex HPV14 assay (Seegene, Werfe, Germany) in an automated real-time multiplex PCR platform, which identifies the following oncogenic genotypes: HPV-16, -18, -31, -33, -35, -39, -45, -51, -52, -56, -58, -59, -66, and − 68^15^. All samples were collected by trained healthcare professionals and not self-administered.

### High-resolution anoscopy (HRA)

According to our institutional screening protocol, referral for high-resolution anoscopy (HRA) was indicated for patients with abnormal cytology—defined as ASCUS, LSIL, HSIL or SCC according to the Bethesda System—as well as for individuals with normal cytology who tested positive for HPV-16 or HPV-18. However, due to limited availability of HRA and variable adherence in routine clinical practice, not all patients meeting referral criteria ultimately underwent the procedure.

HRA was performed by internal medicine specialists trained in the technique, following international clinical guidelines^16^. Acetic acid and Lugol’s iodine were used to visualize intraepithelial squamous lesions (SIL), which were subsequently biopsied. Cases with histological diagnoses of anal intraepithelial neoplasms 2–3 (AIN2–AIN3) (HGAIN) or anal squamous cell carcinoma were referred to the Proctology Unit for surgical ablation and follow-up within the anal dysplasia screening program.

### Collected variables

Clinical and epidemiological data were obtained from the eVIHA electronic medical record. The following variables were recorded: age, smoking status, clinical stage of HIV infection (according to CDC criteria), duration of antiretroviral therapy (ART), time since HIV diagnosis, history of anogenital warts, and prior diagnosis of carcinoma. Laboratory data included nadir CD4, current CD4 count, and HIV viral load at inclusion.

### Statistical analysis

Frequency distributions (number and percentages) and measures of central tendency (mean and standard deviation) were used to describe the study population and the main variables.

Group comparisons (patients with vs. without bacterial STIs, and patients with vs. without HPV infection) were performed using the χ² test or Fisher’s exact test for categorical variables, as appropriate. The Student’s t-test was used to compare continuous variables between groups, while the Mann–Whitney U test was applied to compare the number of HPV genotypes between groups.

The prevalence of different HPV genotypes was calculated as absolute numbers and percentages of the total samples, excluding those with unsatisfactory cytological results.

A p-value < 0.05 was considered statistically significant. Statistical analyses were conducted using Python in Google Colab.

## Results

### Cohort characteristics

During the study period, 715 anal screenings for HGAIN and bacterial STIs were performed in a cohort of 377 PLHIV, comprising 370 (98%) MSM and 7 (1.8%) TW. From 2020, 438 anal screenings were performed.

Each participant underwent a median of two screening visits. The mean age of the cohort was 44 years (SD 11), and 89% (*n* = 338) had an undetectable HIV viral load (< 50 copies/mL) at the time of screening. The mean current CD4 was 771 cells/mL (SD 318). The clinical and epidemiological characteristics of the patients are summarized in Table 1.

**Table 1:** Baseline characteristics from the 377 MSM and TW in the screening program since June 2017. Results are expressed as N(%) unless otherwise specified.

Characteristic	*N* (%) or mean (SD)
Age (years), mean (SD)	44 (11)
Years since HIV diagnosis, mean (SD)	14 (8)
Years on ART, mean (SD)	11 (7)
HIV RNA VL < 50 copies/mL	338 (89%)
HIV RNA VL < 200 copies/mL	356 (94%)
Nadir CD4 T-cell count < 200 cel/µL	60 (15.8%)
Current CD4 T-cell count, mean (SD)	771 (318)
CDC C3 category	35 (9.5%)
Smoking status	100 (26.5%)
HPV vaccination	21 (5.6%)
Previous neoplasia^a^	41 (10.9%)
Previous condylomas	59 (15.6%)

MSM: men who have sex with men; TW: transgender women; VL: viral load; ART: antiretroviral treatment; HPV: human papillomavirus.

^a^Neoplasia not related to anal cancer.

### Prevalence of asymptomatic bacterial anal STIs

**Fig. 1:**
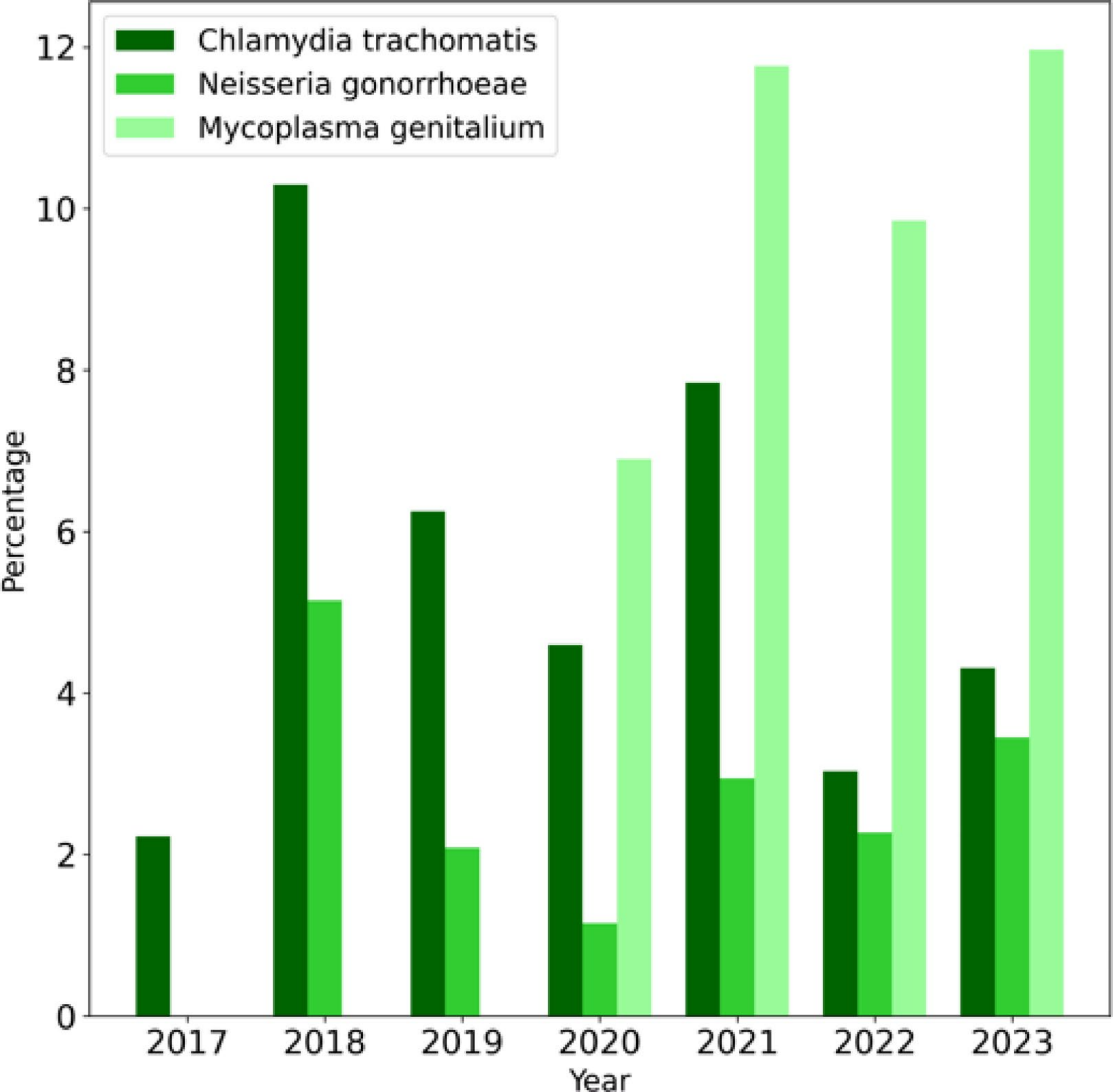
Annual distribution (percentage) of bacterial sexually transmitted infections.

Throughout the study period, 42/715 cases (5.9%) of *C. trachomatis*, 20/715 cases (2.8%) of *N. gonorrhoeae*, and 45/438 cases (10.3%) of *M. genitalium* infection were diagnosed. The annual distribution of these infections is shown in Fig. 1.

A high prevalence of *C. trachomatis* and *N. gonorrhoeae* was observed in 2018, followed by a progressive decline over the subsequent years, except for a transient increase in 2021. In contrast, *M. genitalium* infections demonstrated a steady upward trend starting in 2020.

The mean age of patients with at least one bacterial STIs was 40 years (SD 9), significantly younger than those without STIs (46 years, SD 11; *p* < 0.001). Recurrent infections were identified in 7/45 cases (15.5%) for *M. genitalium*, 2/20 (10%) for *N. gonorrhoeae*, and 3/42 (7%) for *C. trachomatis*.

### Prevalence of HPV infection in anal samples

**Fig. 2:**
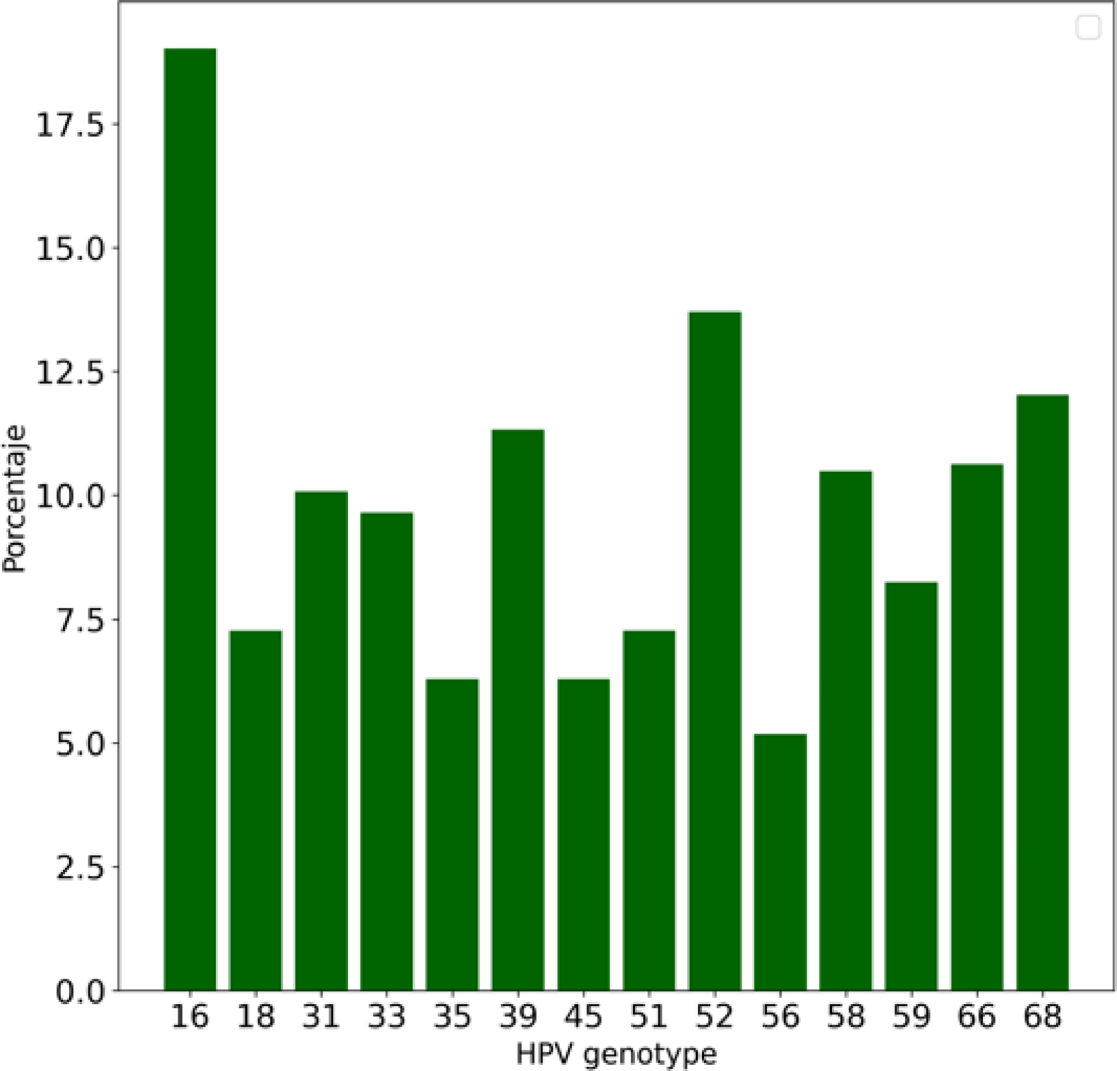
Prevalence of the studied high risk HPV genotypes.

At least one HPV genotype was detected in 397 samples (62.6%), two genotypes in 93 (14.7%), and three or more genotypes in 144 (22.7%). Figure 2 illustrates the prevalence of individual genotypes, with HPV-16 being the most frequent (18%), followed by HPV-52 (14%), HPV-68 (12%), and HPV-39 (11.5%).

### Relationship between bacterial STIs and HPV genotypes

**Fig. 3:**
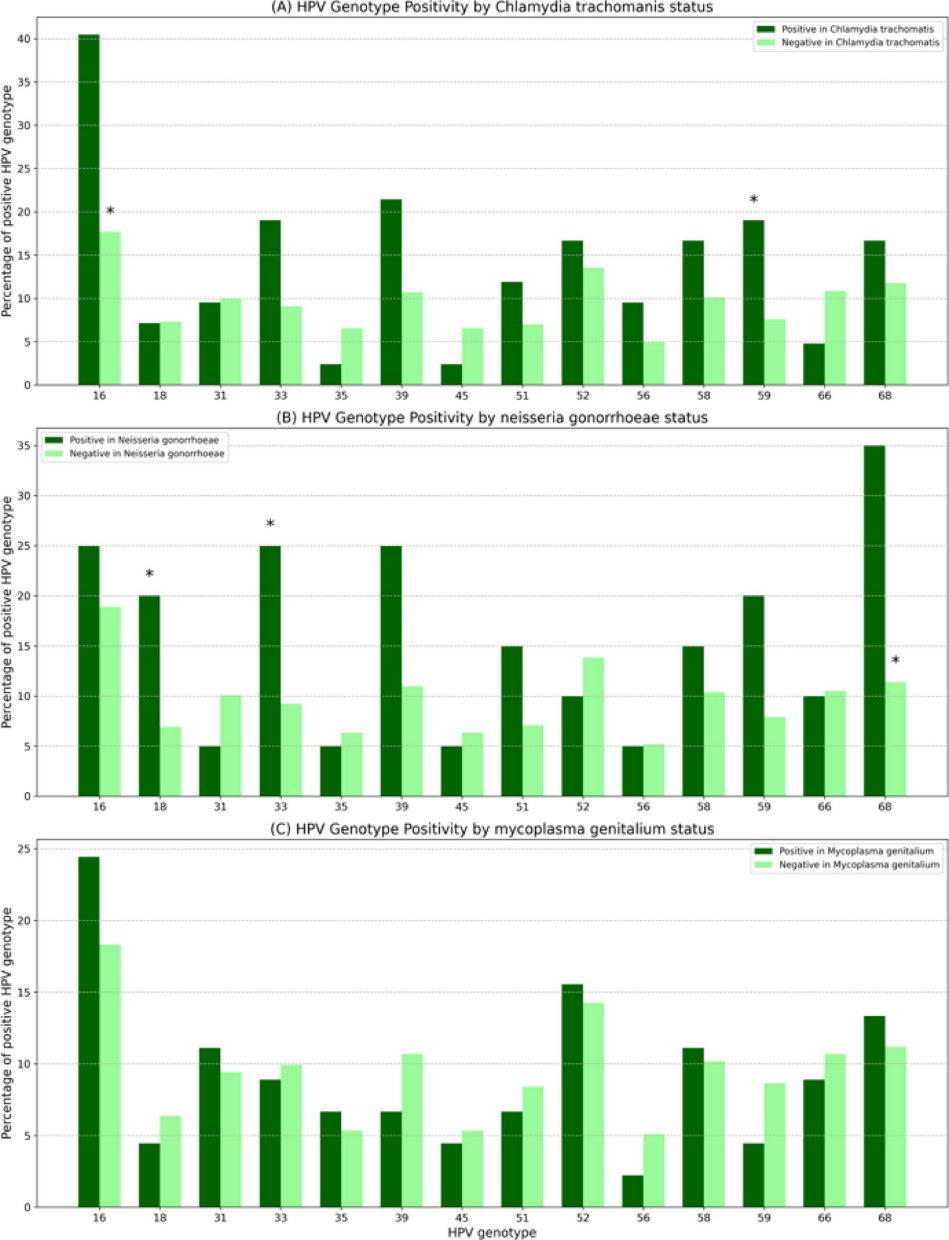
Relationship between the prevalence of HPV genotypes and the presence or absence of bacterial STIs. *Statistically significant at p < 0.05.

When comparing the presence of HPV among participants with and without bacterial STIs, a higher prevalence of HPV-16 (40.5 vs. 17.7%; *p* = 0.0006) and HPV-59 (19.1 vs. 7.6%; *p* = 0.02) was found among those infected with *C. trachomatis*, as shown in Fig. 3A. In patients with *N. gonorrhoeae* infection, HPV-18 (20 vs. 6.9%; *p* = 0.049), HPV-33 (25 vs. 9.2%; *p* = 0.047), and HPV-68 (35 vs. 11.4%; *p* = 0.004) were more frequently detected (Fig. 3B). No specific HPV genotype predominated among those with *M. genitalium* infection (Fig. 3C).

Moreover, a greater diversity of oncogenic genotypes, measured as the number of genotypes present in a particular swab, was found in patients coinfected with *C. trachomatis* or *N. gonorrhoeae*. The median number of HPV genotypes was higher in patients with *C. trachomatis* (median 2 genotypes) or *N. gonorrhoeae* (median 2 genotypes) compared with those without bacterial STIs (median 1 genotype) (p-values 0.008 and 0.009, respectively). This difference was not observed in *M. genitalium*-infected individuals.

### Relationship between bacterial STIs and histological findings

During the study period, 715 cytologies and 91 HRAs with corresponding biopsies were performed. Cytological results were as follows: 490 (68.5%) normal, 87 (12.2%) ASCUS, 46 (6.4%) LSIL, and 10 (1.4%) HSIL.

Histopathological results showed 30 cases (33%) of AIN1, 27 (29.6%) of AIN2–AIN3, and 2 (2.2%) SCC. There was no dysplasia in 32 (35.1%) of them.

When cytology results were compared according to the presence or absence of bacterial STIs (Table 2), no statistically significant differences were observed, although a nonsignificant trend toward a higher frequency of ASCUS cytology was noted among patients with STIs (17 vs. 13.2%). No HSIL cytology results were identified among patients with bacterial STIs.

**Table 2:** Comparison of the cytological and histological results according to the presence or absence of bacterial STI (chlamydia trachomatis, Neisseria gororrhoeae and mycoplasma genitalium).

	Positive bacterial STI	Negative bacterial STI	*p*-value
Cytology results (*N*)	88	545	
Normal	69 (78.4%)	421 (77.3%)	0.917
ASCUS	15 (17.1%)	72 (13.2%)	0.422
LSIL	4 (4.6%)	42 (7.7%)	0.378
HSIL	0 (-)	10 (1.8%)	0.379
Histology results (N)	11	80	
Without dysplasia	2 (18.2%)	30 (37.5%)	0.317
AIN-1	4 (36.4%)	26 (32.5%)	1.000
AIN-2	4 (36.4%)	14 (17.5%)	0.218
AIN-3	1 (9.1%)	8 (10.0%)	1.000
SCC	0 (-)	2 (2.5%)	1.000

ASCUS: atypical squamous cells of undetermined significance; LSIL: low-grade squamous intraepithelial lesion; HSIL: high-grade squamous intraepithelial lesion; AIN: anal intraepithelial neoplasia; SCC: squamous cell carcinoma; STI: sex-transmitted infection.

Similarly, no significant differences were found in biopsy results according to STI status (Table 2).

Furthermore, when comparing cytological and histological outcomes between patients coinfected with both bacterial STIs and high-risk HPV versus those with bacterial STIs alone, no significant associations were observed. However, the proportion of abnormal cytology (ASCUS/LSIL/HSIL) was higher among those with high-risk HPV and bacterial STIs, although this difference did not reach statistical significance (25.1 vs. 12.5%; *p* = 0.25) (Table 3).

**Table 3:** Comparison of the cytological and histological results between coinfected with HPV + bacterial STI (chlamydia trachomatis, Neisseria gororrhoeae and/or mycoplasma genitalium) versus participants with only bacterial STI.

	Coinfection bacterial STI + HPV	Bacterial STI-only patients	*p*-value
Cytology results (*N*)	64	24	
Normal	48 (75.0%)	21 (87.5%)	0.255
ASCUS	12 (18.8%)	3 (12.5%)	0.751
LSIL	4 (6.3%)	0 (-)	0.571
HSIL	0 (-)	0 (-)	-
Histology results (N)	10	1	
Without dysplasia	2 (20%)	0 (-)	0
AIN-1	3 (30%)	1 (100%)	0.364
AIN-2	4 (40%)	0 (-)	1.000
AIN-3	1 (10%)	0 (-)	1.000
SCC	0 (-)	0 (-)	1.000

ASCUS: atypical squamous cells of undetermined significance; LSIL: low-grade squamous intraepithelial lesion; HSIL: high-grade squamous intraepithelial lesion; AIN: anal intraepithelial neoplasia; SCC: squamous cell carcinoma; STI: sex-transmitted infection.

## Discussion

In this prospective study of MSM and TW living with HIV, we found that asymptomatic bacterial anal STIs were frequent and associated with greater diversity of oncogenic HPV genotypes. However, no statistically significant relationship was observed between these infections and the presence of high-grade anal intraepithelial neoplasia (HGAIN). However, although no statistically significant association with HGAIN was demonstrated, the numerically higher frequency of dysplastic cytology among individuals coinfected with high-risk HPV warrants further investigation in longitudinal studies.

The prevalence of Chlamydia trachomatis, Neisseria gonorrhoeae, and *Mycoplasma genitalium* in our population was comparable to that reported in other cohorts of MSM living with HIV, reinforcing the need for periodic screening strategies even in the absence of symptoms^11,17^. Over the past decade, anal STIs among PLHIV—particularly MSM and TW—have shown a sustained increase. Several factors may contribute to this trend, including broader use of HIV pre-exposure prophylaxis (PrEP), which, although highly effective in preventing HIV infection, has raised concerns about its potential impact on bacterial STI incidence^18,19^. Another key aspect is that many of these infections are asymptomatic, which facilitates both persistence and transmission^20,21^.

Among bacterial STIs, *C. trachomatis* has been proposed as a potential cofactor in anal carcinogenesis due to its ability to modulate HPV infection. As an intracellular pathogen, it can induce chronic inflammation, disrupt epithelial integrity, and alter gene expression, potentially promoting both HPV persistence and progression to high-grade intraepithelial lesions^8–10,22,23^. Consistent with this biological rationale, Masiá et al.^12^ demonstrated in a cohort of PLHIV that *C. trachomatis* infection was significantly associated with an increased risk of HGAIN. However, this association has not been consistently replicated. A more recent analysis by Rizzo et al.^13^ did not find a statistically significant relationship between bacterial STIs and anal dysplasia, highlighting the heterogeneity of published results and the need for larger, multicenter studies.

Despite these discrepancies, both Masiá et al.^12^ and Rizzo et al.^13^ reported a direct link between bacterial STIs and oncogenic HPV genotypes—particularly HPV-16—supporting the hypothesis that these coinfections may influence viral persistence and long-term oncogenic potential. In our cohort, we observed a significantly higher prevalence of HPV-16 among individuals infected with *C. trachomatis* compared to those without this infection (40.5% vs. 17.7%; *p* = 0.0006), reinforcing this association. Notably, this pattern was not observed in participants with *N. gonorrhoeae* or *M. genitalium*, suggesting that interactions between bacterial STIs and specific HPV genotypes may be pathogen-dependent.

In our previous analysis of this cohort^4^, infection with multiple oncogenic HPV genotypes was highly prevalent, and HPV-16 showed the strongest association with HSIL, being present in the majority of high-grade lesions. Likewise, large epidemiological studies have consistently confirmed the central role of HPV-16 in anal HSIL among MSM living with HIV, as well as the high burden of multigenotype infections in this population^24,25^. Collectively, these data suggest that detection of multiple oncogenic HPV genotypes may represent a marker of sustained mucosal exposure and reduced viral clearance capacity, potentially increasing the likelihood of persistent infection and progression to high-grade intraepithelial neoplasia. However, longitudinal studies specifically evaluating genotype-specific persistence are required to establish this temporal relationship.

Within this framework, our findings occupy an intermediate position. Although we did not demonstrate a statistically significant association between bacterial STIs and anal dysplasia, patients with STIs exhibited greater diversity of high-risk HPV genotypes and a higher prevalence of HPV detection. This pattern aligns with observations from other cohorts^24,25^ and with our prior findings^4^, and supports the interpretation that bacterial STIs may not act as direct causal factors for HGAIN but rather as indirect modulators of HPV dynamics, potentially contributing to conditions associated with viral persistence.

Another noteworthy finding in our cohort was the steady increase in M. genitalium infections in recent years, particularly following the COVID-19 pandemic. This rise may reflect changes in sexual behavior during the post-pandemic period and therapeutic challenges in managing asymptomatic infections, both of which could contribute to persistence and transmission. Although no direct evidence currently links *M. genitalium* to anal dysplasia, several studies have explored its association with HPV infection and cervical lesions, suggesting a potential role in mucosal carcinogenesis^26,27^.

Finally, our results reaffirm that among PLHIV, younger and sexually active MSM and TW represent the population at highest risk for bacterial STI acquisition. This underscores the importance of implementing integrated prevention and screening strategies that include not only HPV detection but also bacterial STI surveillance, as part of a comprehensive approach to sexual health care in this high-risk population.

Our study has several limitations. First, the relatively low number of biopsies confirming HGAIN reduces the statistical power to establish robust associations between bacterial STIs and high-grade lesions. Second, although referral for high-resolution anoscopy (HRA) was protocolized for patients with abnormal cytology and/or HPV-16/18 positivity, limited access to HRA and variable patient adherence in routine clinical practice meant that not all eligible individuals underwent the procedure. This real-world constraint may have introduced verification bias and could have led to an underestimation of the true prevalence of high-grade lesions. Third, although anal dysplasia screening was systematically offered to all eligible MSM and transgender women living with HIV at our unit, formal data regarding acceptance or refusal rates were not systematically recorded, and participation was voluntary. Fourth, sexual behaviour was not assessed using a standardized questionnaire during the study period; therefore, residual confounding related to sexual practices cannot be excluded. Fifth, the late incorporation of *M. genitalium* testing (from 2020 onward) may have underestimated its true prevalence. Finally, as this was a single-center study, the generalizability of our findings should be approached with caution. Nevertheless, the real-world design of our screening program reflects the practical challenges of implementing anal dysplasia surveillance in routine clinical care and provides clinically relevant insight into the feasibility and limitations of such strategies in high-risk populations.

Despite these limitations, our data provide valuable evidence on the interaction between bacterial STIs and HPV in PLHIV. The observed association between bacterial infections and greater diversity of oncogenic HPV genotypes highlights the potential value of integrating bacterial STI screening into a comprehensive sexual health strategy for PLHIV. Multicenter, longitudinal studies are warranted to clarify the clinical significance of these interactions and to determine whether bacterial STIs influence HPV persistence or the development of high-grade anal lesions.

In conclusion, asymptomatic bacterial anal STIs were common among MSM and TW living with HIV and were associated with increased diversity of oncogenic HPV genotypes. Although no significant relationship with HGAIN was demonstrated, our findings suggest that bacterial STIs may influence HPV genotype distribution and viral dynamics in this high-risk population. Further longitudinal studies are needed to determine whether these interactions have implications for the development of high-grade anal lesions.

These findings support consideration of bacterial STI screening within anal dysplasia monitoring protocols for PLHIV as part of a broader, integrated approach to sexual health care in this high-risk population.

## Data Availability

The datasets used and/or analysed during the current study, as well as all the code, are available from the corresponding author on reasonable request.

## Abbreviations

AIN: Anal intraepithelial neoplasia
ART: Antiretroviral treatment
ASCUS: Atypical squamous cells of undetermined significance
HGAIN: High grade anal intraepithelial neoplasia
HSIL: High-grade squamous intraepithelial lesion
HPV: Human papillomavirus
LSIL: Low-grade squamous intraepithelial lesion
MSM: Men who have sex with men
PLHIV: People living with HIV
SCC: Squamous cell carcinoma
STI: Sex-transmitted infection
TW: Transgender women
VL: Viral load

## References

[1] Guisado, Y. M. et al. Incidence rate and risk factors for anal squamous cell carcinoma in a cohort of people living with HIV from 2004 to 2017: implementation of a screening program. *Dis. Colon Rectum*. **65**, 28–39 (2022). 34694279 10.1097/DCR.0000000000002218

[2] Burgos, J. et al. Risk of progression to high-grade anal intraepithelial neoplasia in HIV-infected MSM. *AIDS* **29**, 695–702 (2015). 25849833 10.1097/QAD.0000000000000603

[3] McCutcheon, T. et al. Progression of anal intraepithelial neoplasia in HIV-positive individuals: predisposing factors. *Tech. Coloproctol*. **23**, 325–332 (2019). 31016550 10.1007/s10151-019-01951-w PMC6582960

[4] Villoslada, A. et al. The role of human papillomavirus genotypes on anal squamous intraepithelial lesions among gay, bisexual and other men who have sex with men living with HIV: beyond HPV-16. *J. Med. Virol.* **97**, e70596 (2025). 40923874 10.1002/jmv.70596 PMC12419139

[5] Shiels, M. S. et al. Projected cancer incidence rates and burden of incident cancer cases in HIV-infected adults in the United States through 2030. *Ann. Intern. Med.* **168**, 866–873 (2018). 29801099 10.7326/M17-2499 PMC6329294

[6] Hidalgo-Tenorio, C. et al. Risk factors for high-grade anal intraepithelial lesions in MSM living with HIV and the response to topical and surgical treatments. *PLoS One*. **16**, e0245870 (2021). 33534790 10.1371/journal.pone.0245870 PMC7857605

[7] Daling, J. R. et al. Human papillomavirus, smoking, and sexual practices in the etiology of anal cancer. *Cancer* **101**, 270–280 (2004). 15241823 10.1002/cncr.20365

[8] Kumari, S. & Bhor, V. M. A literature review on correlation between HPV coinfection with Chlamydia trachomatis and cervical neoplasia. *Microb. Pathog*. **168**, 105587 (2022). 35588965 10.1016/j.micpath.2022.105587

[9] Koskela, P. et al. Chlamydia trachomatis infection as a risk factor for invasive cervical cancer. *Int. J. Cancer*. **85**, 35–39 (2000). 10585579 10.1002/(sici)1097-0215(20000101)85:1<35::aid-ijc6>3.0.co;2-a

[10] Jensen, K. E. et al. Chlamydia trachomatis and risk of cervical intraepithelial neoplasia grade 3 or worse in women with persistent human papillomavirus infection: a cohort study. *Sex. Transm Infect.* **90**, 550–555 (2014). 24728044 10.1136/sextrans-2013-051431

[11] Zhang, Q. et al. High rates of Treponema pallidum, Neisseria gonorrhoeae, Chlamydia trachomatis, or Trichomonas vaginalis co-infection in people with HIV: a systematic review and meta-analysis. *Eur. J. Clin. Microbiol. Infect. Dis.* **44**, 1–15 (2025). 39466544 10.1007/s10096-024-04966-w

[12] Masiá, M. et al. Infection with Chlamydia trachomatis increases the risk of high-grade anal intraepithelial neoplasia in people living with human immunodeficiency virus. *Clin. Infect. Dis.* **70**, 2161–2167 (2020). 31271192 10.1093/cid/ciz606

[13] Rizzo, A. et al. Anal HPV prevalence in individuals with and without other concomitant sexually transmitted infections. *J. Med. Virol.* **96**, e29852 (2024). 39166456 10.1002/jmv.29852

[14] Solomon, D. et al. The 2001 Bethesda system: terminology for reporting results of cervical cytology. *JAMA* **287**, 2114–2119 (2002). 11966386 10.1001/jama.287.16.2114

[15] Lee, D. H. et al. Comparison of the performance of Anyplex II HPV HR, the Cobas 4800 human papillomavirus test and Hybrid Capture 2. *Ann. Clin. Biochem.* **53**, 561–567 (2016). 26486441 10.1177/0004563215614036

[16] Palefsky, J. M. Practising high-resolution anoscopy. *Sex. Health*. **9**, 580–586 (2012). 23380236 10.1071/SH12045

[17] Ooi, C., Kong, F. Y. S., Lewis, D. A. & Hocking, J. S. Prevalence of sexually transmissible infections and HIV in men attending sex-on-premises venues in Australia: a systematic review and meta-analysis of observational studies. *Sex. Health*. **17**, 135–148 (2020). 32228828 10.1071/SH19150

[18] Jansen, K. et al. STI in times of PrEP: high prevalence of chlamydia, gonorrhea, and mycoplasma at different anatomic sites in men who have sex with men in Germany. *BMC Infect. Dis.* **20**, 110 (2020). 32033533 10.1186/s12879-020-4831-4 PMC7007644

[19] Ong, J. J. et al. Global epidemiologic characteristics of sexually transmitted infections among individuals using preexposure prophylaxis for the prevention of HIV infection: a systematic review and meta-analysis. *JAMA Netw. Open.* **2**, e1917134 (2019). 31825501 10.1001/jamanetworkopen.2019.17134 PMC6991203

[20] Schumacher, C. et al. Sexually transmitted infection screening among gay, bisexual, and other men who have sex with men prescribed pre-exposure prophylaxis in Baltimore City, Maryland. *Clin. Infect. Dis.* **71**, 2637–2644 (2020). 31761944 10.1093/cid/ciz1145

[21] Malekinejad, M. et al. Risk of HIV acquisition among men who have sex with men infected with bacterial sexually transmitted infections: a systematic review and meta-analysis. *Sex. Transm Dis.* **48**, e138–e148 (2021). 33783414 10.1097/OLQ.0000000000001403 PMC8485981

[22] Zadora, P. K. et al. Integrated phosphoproteome and transcriptome analysis reveals Chlamydia-induced epithelial-to-mesenchymal transition in host cells. *Cell. Rep.* **26**, 1286–1298 (2019). 30699355 10.1016/j.celrep.2019.01.006

[23] Arcia Franchini, A. P. et al. The role of Chlamydia trachomatis in the pathogenesis of cervical cancer. *Cureus* **14**, e21331 (2022). 35186589 10.7759/cureus.21331 PMC8849235

[24] Hernandez, A. L. et al. Prevalence of anal human papillomavirus infection and anal high-grade squamous intraepithelial lesions among men who have sex with men 50 years and older living with or without HIV. *J. Acquir. Immune Defic. Syndr.* **96**, 439–446 (2024). 38985441 10.1097/QAI.0000000000003450 PMC11444595

[25] Wei, F. et al. Epidemiology of anal human papillomavirus infection and high-grade squamous intraepithelial lesions in 29,900 men according to HIV status, sexuality, and age: a collaborative pooled analysis of 64 studies. *Lancet HIV*. **8**, e531–e543 (2021). 34339628 10.1016/S2352-3018(21)00108-9 PMC8408042

[26] Ye, H. et al. Association between genital mycoplasmas infection and human papillomavirus infection, abnormal cervical cytopathology, and cervical cancer: a systematic review and meta-analysis. *Arch. Gynecol. Obstet.* **297**, 1377–1387 (2018). 29520664 10.1007/s00404-018-4733-5

[27] Plisko, O. et al. Prediction of high-grade cervical precancerous abnormalities: the role of personal factors, vaginal microflora, sexually transmitted infections, and high-risk human papillomavirus. *PLoS One*. **19**, e0313004 (2024). 39527583 10.1371/journal.pone.0313004 PMC11554082

